# VCE-004.8, A Multitarget Cannabinoquinone, Attenuates Adipogenesis and Prevents Diet-Induced Obesity

**DOI:** 10.1038/s41598-018-34259-0

**Published:** 2018-10-31

**Authors:** Belen Palomares, Francisco Ruiz-Pino, Carmen Navarrete, Inmaculada Velasco, Miguel A. Sánchez-Garrido, Carla Jimenez-Jimenez, Carolina Pavicic, Maria J. Vazquez, Giovanni Appendino, M. Luz Bellido, Marco A. Calzado, Manuel Tena-Sempere, Eduardo Muñoz

**Affiliations:** 1 Instituto Maimónides de Investigación Biomédica de Córdoba (IMIBIC), Córdoba, Spain; 2 Departamento de Biología Celular, Fisiología e Inmunología, Universidad de Córdoba, Córdoba, Spain; 3 Hospital Universitario Reina Sofía, Córdoba, Spain; 4 Vivacell Biotechnology España, Córdoba, Spain; 5 Innohealth Group, Madrid, Spain; 6 Dipartimento di Scienze del Farmaco, Università del Piemonte Orientale, Novara, Italy; 7 Emerald Health Pharmaceuticals, San Diego, USA

## Abstract

Over the past few years, the endocannabinoid system (ECs) has emerged as a crucial player for the regulation of food intake and energy metabolism, and its pharmacological manipulation represents a novel strategy for the management of metabolic diseases. The discovery that VCE-004.8, a dual PPARγ and CB_2_ receptor agonist, also inhibits prolyl-hydroxylases (PHDs) and activates the HIF pathway provided a rationale to investigate its effect in *in vitro* models of adipogenesis and in a murine model of metabolic syndrome, all processes critically regulated by these targets of VCE-004.8. In accordance with its different binding mode to PPARγ compared to rosiglitazone (RGZ), VCE-004.8 neither induced adipogenic differentiation, nor affected osteoblastogenesis. Daily administration of VCE-004.8 (20 mg/kg) to HFD mice for 3-wks induced a significant reduction in body weight gain, total fat mass, adipocyte volume and plasma triglycerides levels. VCE-004.8 could also significantly ameliorate glucose tolerance, reduce leptin levels (a marker of adiposity) and increase adiponectin and incretins (GLP-1 and GIP) levels. Remarkably, VCE-004.8 increased the FGF21 mRNA expression in white and brown adipose, as well as in a BAT cell line, qualifying cannabinoaminoquinones as a class of novel therapeutic candidates for the management of obesity and its common metabolic co-morbidities.

## Introduction

Obesity and metabolic syndrome (MetS) are interconnected conditions whose prevalence is growing at an alarming rate worldwide. Indeed, while WHO estimated that in 2005 ~300 million people had a BMI ≥ 30 kg/m^2^, by 2014 > 600 million adults (13% of total population) were obese (see WHO, Global status report on non-communicable diseases 2014, document number WHO/NMH/NVI/15.1, accessible at www.who.int/mediacentre/). Central obesity is the fundamental contributing factor for MetS, whose mean prevalence ranges between 20–25% of the world population, with variations depending on the geography, ethnicity, age and sex^1,2^. MetS causes >5-fold increase in the risk of type-2 diabetes (T2D) and >2-fold rise in the risk of developing cardiovascular disease, as well as in all-cause mortality^2,3^. Notably, the phenotypic presentation of MetS and its clinical evolution (e.g., in terms of co-morbidities) is rather variable; MetS being a heterogeneous condition, whose pathophysiological basis, which is likely multifaceted^4,5^, remains ill defined.

Over the past few decades, the endocannabinoid system (ECs) has emerged as a pivotal component of the homeostatic mechanisms for the control of body weight and metabolism^6,7^. This system integrates endocannabinoids such as anandamide (AEA) and 2-arachidonoyl-glycerol (2-AG), their receptors (CB_1_ and CB_2_), and the enzymatic machinery for their synthesis and metabolic inactivation^8^. CB receptors are also targeted by natural and synthetic cannabinoids, which mimic (or antagonize) the effects of EC. Notably, while CB_1_ is widely expressed in the brain, as well as in peripheral tissues, and has been unambiguously related to circuits governing energy balance and metabolic homeostasis^6^, CB_2_ has a predominant peripheral expression, and is mostly present in immune cells and involved in modulation of inflammatory responses^7^. Obesity and MetS have been defined as conditions of over-activation of the ECs, and therapies based on reverse-agonism of CB_1_ were proven effective to ameliorate the metabolic complications of obesity. Nevertheless, adverse neurological effects related to CB_1_-mediated central actions led to their demise^6,7^. Nevertheless, strategies targeting the peripheral actions of EC still hold promise for the management of MetS, being devoid of the adverse central actions of the CB_1_ reverse-agonists^7,9^, but the precise role for of CB_2_ in mediating the metabolic actions of EC remains unclear.

The peroxisome proliferator-activated receptor-γ (PPARγ) is a nuclear receptor that plays key role in regulating a large number of biological functions including lipid metabolism and glucose homeostasis^10^. PPARγ ligands include a wide array of natural and synthetic molecules, among which the best characterized are glitazones, as exemplified by rosiglitazone (RGZ), which has been extensively used in patients with type-2 diabetes. However, full agonists activators (PPARγ-fa) have undesirable side effects like weight gain, edema, liver injury, cancer, as well as an increased risk of heart failure^11^. Furthermore, reduction of bone mass and an increased risk of peripheral fractures in glitazone-treated patients have also been observed and associated to the inhibition of bone marrow osteoblastogenesis^12^. Thus, PPARγ controls bone mass through differentiation of MSCs toward osteoblasts and adipocytes, and RGZ suppresses osteoblast and promotes adipocyte development^13^. More recently, it has been shown that RGZ stimulates osteoblast differentiation in human MSCs, but this differentiation was followed by oxidative stress and apoptosis, overall resulting in a net loss of osteoblasts in the bone marrow^14^. Therefore, while the physiologic and therapeutic potential of PPARγ modulation remains high, interest has substantially shifted towards partial ligands, and cannabinoid-type molecules have raised considerable interest as safer alternatives to PPARγ-fa for anti-diabetic drug candidates^15^.

Studies on the pathogenic mechanisms of metabolic disease have documented that obesity is a chronic hypoxic state^16^ that triggers adaptive responses mediated by hypoxia-inducible factor (HIF)-1α and HIF-2α and aimed at restoring oxygen homeostasis^16,17^. The mechanism by which oxygen controls HIF-1α and HIF-2α stabilization has been clarified by the identification of prolyl-hydroxylases (PHDs), non-heme Fe(II) dioxygenases that require molecular oxygen and 2-oxoglutarate to hydroxylate HIF-1α and HIF-2α. Under normoxic conditions, hydroxylated HIF is ubiquitinated by an E3-ubiquitin ligase and targeted for degradation by the 26S proteasome^18^. Despite a plethora of studies addressing the roles of HIFs in adipose dysfunction^19–22^, the involvement of HIF-1α and HIF-2α in obesity remains controversial. On one hand, hypoxia is thought to exacerbate macrophage-mediated inflammation in obesity, and activation of the HIF pathway might contribute to the obese phenotype. However, other studies have shown that hypoxia actually promotes body weight reduction. In fact, hypomorphic mice for PHD2 (Hif-p4h-2), where the expression of PHD2 is significantly reduced in different tissues, showed cardioprotection via induction of the expression of genes involved in glucose metabolism, cardiac function and blood pressure. In addition, the weight of Hif-p4h-2 mice was significantly lower than that of wild-type mice^23^. Subsequent studies have shown that Hif-p4h-2 mice have less adipose tissue, higher glucose tolerance, better insulin sensitivity, lower plasma cholesterol levels and are protected against HFD-induced hepatic steatosis^24^, and similar results were observed with the pharmacological inhibition of PHD2. Moreover, erythropoietin, whose gene is regulated by HIF, has been shown to prevent adipogenesis and improve obesity, insulin resistance and adipose tissue inflammation in HFD mice^25,26^. These findings have provided a rationale for the evaluation of PHD-HIF modulators in the management of MetS.

In our search for multi-target compounds of potential therapeutic utility, we have documented the beneficial effects of cannabinoquinones in chronic degenerative diseases. In particular, the cannabidiol aminoquinone VCE-004.8 was shown to possess dual CB_2_ and PPARγ agonism, also inhibiting PHD1 and PHD2 activities and activating the HIF pathway^27,28^. This pharmacological profile makes VCE-004.8 of interest for the management of obesity and MetS, and we present data that validate this assumption.

## Results

### VCE-004.8 is a selective PPARγ modulator

**Figure 1:**
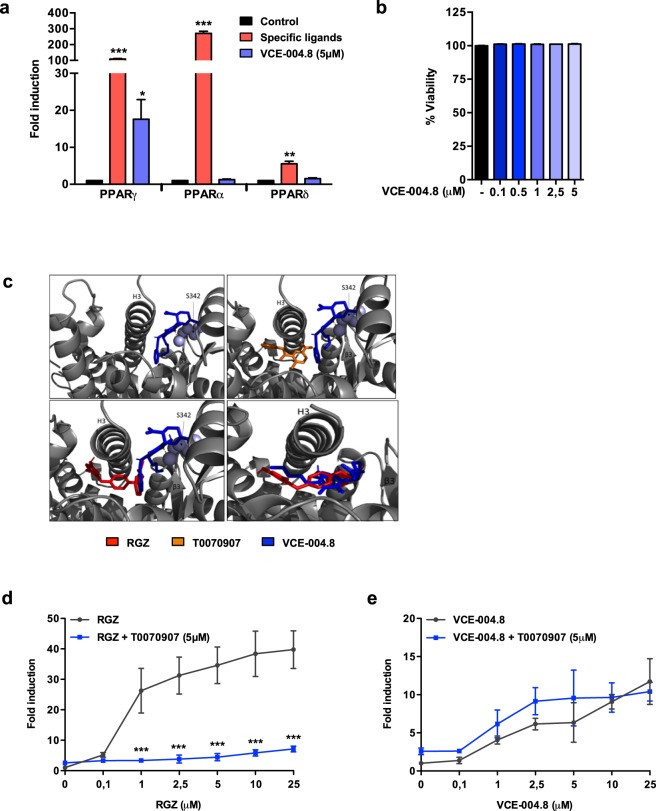
Characterization of VCE-004.8 as a selective PPARγ agonist. **(a)** Receptor-specific transactivation by VCE-004.8. HEK-293T cells were co-transfected with a GAL4-luc reporter and GAL4-PPARγ, GAL4-PPARα and GAL4-PPARδ and treated with VCE-004.8 (5 μM) for 6 hours and luciferase activity measured in the cell lysates. Results are expressed as the fold induction ± SD (n = 3) relative to untreated control. Control (black bars), VCE-004.8 (blue bars) and specific ligands for each receptor (red bars): RGZ (5 μM) for PPARγ, WY14643 (5 μM) for PPARα, and GW0742 (5 μM) for PPARδ. Results are shown as mean ± S.D. (**b**) Cytotoxic activity of VCE-004.8. HEK-293T cells were treated with the compounds at the indicated concentrations for 6 hours and cell viability was analyzed by the YOYO-1 method. Results are shown as mean ± SD and expressed as percentage of cell viability (n = 3). **(c)** PPARγ LBD structure 3B0R bound to VCE-004.8 (blue) with and without of T0070907 (orange) or RGZ (red). PPARγ LBD structure 4EMA bound to VCE-004.8 and RGZ (lower right panel). **(d**,**e)** PPARγ transcriptional activity of VCE-004.8 and RGZ in the presence and the absence of T0070907 is shown. HEK-293T cells were co-transfected with GAL4-PPARγ and GAL4-luc, pretreated with T0070907 for 15 min and then stimulated with increasing concentrations of either RGZ or VCE-004.8 for 6 hours and luciferase activity measured. Results are expressed as the fold induction ± SD relative to RGZ (D) or VCE-004.8 (E) (n = 4). ^*^P < 0.05, ^**^P < 0.01 and ^***^P < 0.001 agonist ligands or VCE-004.8 treatment vs. control or RGZ + T0090709 vs. RGZ. (ANOVA followed by Turkey’s test or unpaired two-tailed Student’s t-test).

We have previously found that VCE-004.8 binds and activates PPARγ^28^, and were interested to analyze whether or not this compound was able to activate other PPAR family members. We found that VCE-004.8, at non-toxic concentrations, selectively induced PPARγ-dependent transcriptional activity, although with lower potency than RGZ (PPARγ: *p* < 0.001 RGZ vs untreated; *p* = 0.0407 VCE-004.8 vs untreated; Fig. 1a,b), suggesting that VCE-004.8 is a selective agonist of the PPARγ isoform.

PPARγ has a large ligand-binding pocket (LBP), and the diversity of modes in which ligands can be accommodated is associated to distinct biological profiles. The LBP extends from the C-terminal helix H12 to the β-sheet S1/S2 and is divided into AF-2 and β-sheet sub-pockets^29^. Based on different PPARγ complex structures, it has been proposed that full agonists, such as RGZ, bind to both sub-pockets, establishing hydrogen bonds with residues Tyr473 (H12) on AF-2 (also called canonical binding site) and Ser342 (S1/S2) on β-sheet sub-pocket (also called alternative binding site), whereas partial agonists only significantly bind to the alternate site^30,31^. Docking experiments based on the crystal structures 3B0R, 4EMA, 5Y20 and 5LGS deposited in the Protein Data Bank (PDB) were carried out on VCE-004.8. As depicted in Fig. 1c, molecular docking with 3B0R indicated that in the absence of T0070907, an irreversible PPARγ antagonist covalently binding to Cys285 into the PPARγ LBP canonical binding site, VCE-004.8 binds to Ser342 in Ω-loop β3 (alternative site) with a predicted *Ki* of 448.03 nM. Docking analysis using 4EMA and 5Y20 predicts a similar binding pattern, with calculated *Ki* of 95.59 nM and 67.68 nM, respectively. In addition, VCE-004.8 also showed a predicted binding to I218 in Helix 3, rationalizing its higher affinity to crystal 5Y20. Docking analysis (3B0R) in the presence of T0070907 and RGZ, which bind to the canonical site, did not displace the hydrogen bonding interaction of VCE-004.8 with Ser342 (Fig. 1c), rather enhancing the binding its affinity to the alternative site (*Ki* 236.22 nM in the presence of T0070907 and *Ki* 121.55 nM in the presence of RGZ). Interestingly, in the presence of RGZ, VCE-004.8 could also interact with the canonical binding site, suggesting that VCE-004.8 could, in principle, mediate biological activities through both the canonical and the alternative LBP PPARγ sites. To assess this, the potential functionality of the canonical and alternative PPARγ sites involved in the response to VCE-004.8 were investigated. Luciferase reporter assays were used to study the participation of the canonical and alternative binding sites in the response to VCE-004.8 in comparison with RGZ. As expected, pre-incubation with the selective inhibitor of the canonical PPARγ site, T0070907, effectively blocked RGZ-induced PPARγ transactivation (*p* < 0.001 RGZ + T0070907 vs RGZ; Fig. 1d). Conversely, T0070907 did not block VCE-004.8-induced PPARγ transcriptional activity (Fig. 1e). These findings are consistent with the fact that RGZ activates PPARγ by acting mainly, but not exclusively, through the canonical binding site^31^.

### Effect of VCE-004.8 on adipogenic and osteoblastogenic differentiation

**Figure 2:**
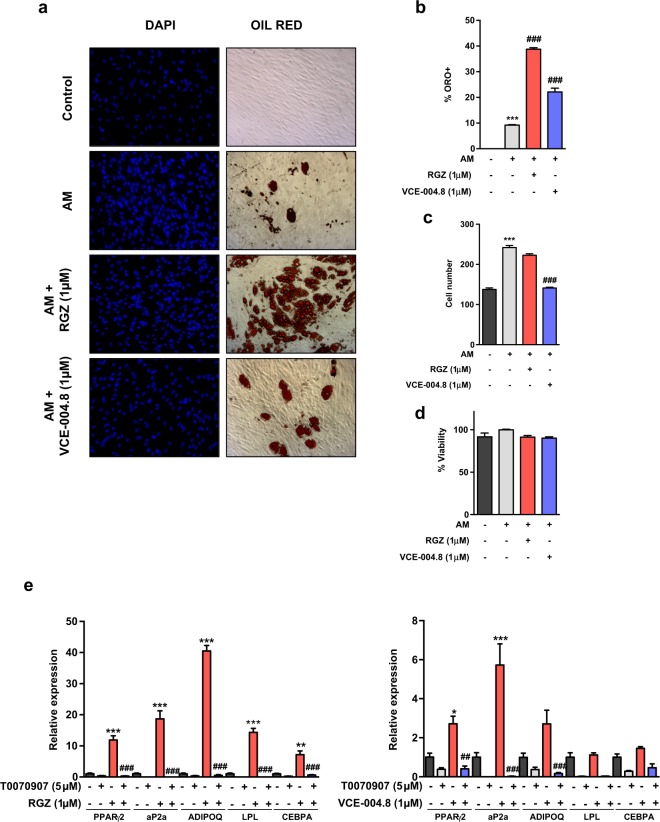
Effect of VCE-004.8 on MSCs differentiation. MSCs were differentiated in adipogenic medium (AM) in the presence of RGZ or VCE-004.8. (**a**) Representative images of Oil Red O (ORO) staining and DAPI nuclear counterstain of MSCs undergoing adipogenic differentiation. Quantification of Oil Red O positive cells (**b**) and DAPI nuclear cells (**c**) after 21 days of differentiation is presented. (**d**) Cytotoxicity of RGZ and VCE-004.8 was evaluated by the MTT method. (**e**) MSCs were differentiated in AM with RGZ or VCE-004.8 in the presence and the absence of T0070907 for 7 days and gene expression of adipogenic markers measured by qPCR. Results represent the mean ± S.D (n = 3). For (B, C) ^***^P < 0.001 AM vs. control; ^###^P < 0.001 RGZ or VCE-004.8 + AM vs. AM; for (E) ^*^P < 0.05, ^**^p < 0.01 and ^***^P < 0.001 RGZ or VCE-004.8 vs. the control cells; ^#^P < 0.05, ^##^P < 0.01 and ^###^P < 0.001 RGZ or VCE-004.8 + T0070907 vs. RGZ or VCE-004.8. (ANOVA followed by Turkey’s test).

Our initial results strongly suggested that, in contrast to RGZ, VCE-004.8 is a PPARγ partial ligand agonist. Since PPARγ is a master regulator of adipogenesis^32^, we studied the ability of VCE-004.8 to influence MSCs differentiation into adipocytes. To this purpose, MSCs were cultured in adipogenic medium (AM) for either 7 days or 21 days to study, respectively, the mRNA expression of adipogenic markers or detect lipid droplets. In the event, hMSC treated with VCE-004.8 showed fewer and smaller lipid droplets (Fig. 2a,b). In addition, VCE-004.8 prevented mitotic expansion of hMSCs (Fig. 2c) and the reduction in the number of cells was not caused by cytotoxicity (Fig. 2d). VCE-004.8 induced lower expression of the adipogenic differentiation markers PPARγ, aP2a, ADIPOQ, LPL and CEBPA (PPARγ2: *p* = 0.0415; aP2a: *p* < 0.0001; Fig. 2e) as compared to cells treated with the PPARγ-fa, RGZ (PPARγ2: *p* < 0.0001; aP2a: *p* < 0.0001; ADIPOQ: *p* < 0.0001; LPL: *p* < 0.0001; CEBPA: *p* < 0.0001; Fig. 2e). Interestingly, the effect of VCE-004.8 on adipocyte differentiation was prevented by T0070907 (PPARγ2: *p* = 0.0012; aP2a: *p* < 0.0001; ADIPOQ: *p* < 0.0003; Fig. 2e), suggesting that this PPARγ ligand can signal through both the canonical and alternative LBP binding site (Fig. 2e compared to Fig. 1e).

There is evidence that glitazones, like RGZ, suppress MSC osteoblast development through the PPARγ pathway, mechanistically rationalizing the observation of bone loss after prolonged use of this class of drugs^33^. However, other authors have shown that RGZ does not interfere directly with osteoblastogenesis in hMSCs^14^. Nevertheles, we found that VCE-004.8 neither inhibited osteoblast mineralization, nor suppressed the expression of osteogenic differentiation markers, such as Runx2 and ALP, in hMSC differentiated in an osteoblastogenic medium (OM) for 21 days (Supplementary Fig. 1). Altogether, these data indicate that VCE-004.8 qualifies as a partial PPARγ ligand, being significantly less adipogenic than RZG and not interfering with osteoblasts differentiation.

### VCE-004.8 treatment ameliorates HFD-induced metabolic perturbations

**Figure 3:**
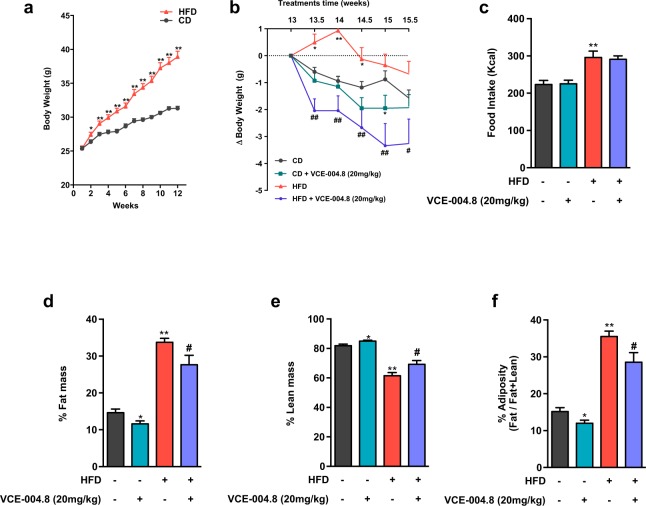
Effect of VCE-004.8 on body weight gain, food intake and body composition. (**a**) Body weight (BW) curves of adult male mice fed for 15-weeks with high fat diet (HFD) or the corresponding control diet (CD). (**b**) BW gain (g) in HFD and CD mice treated for the last three weeks with VCE-004.8 or vehicle; values are referenced to BW at the beginning of treatment (taken as 0). (**c**) Total calorie intake (Kcal) during the treatment period in HFD and CD mice injected with VCE-004.8 or vehicle. In addition, the percentage fat and lean mass, as well as percentage of adiposity, at the end of treatments are presented in (**d**–**f**) for the four experimental groups. Values correspond to means ± SEM of at least 8 mice per group. **P < 0.01 and ^***^P < 0.001 VCE-004.8-treated mice or HFD mice vs. control (CD) mice; ^#^P < 0.05 and ^##^P < 0.01 VCE-004.8-treated HFD mice vs. HFD mice treated with vehicle (ANOVA followed by Turkey’s test or unpaired two-tailed Student’s t-test).

To evaluate the potential beneficial effects of VCE-004.8 on metabolic disease, we studied the impact of chronic administration of this compound in a mouse model of HFD-induced obesity, which displays features of MetS. HFD exposure for up to 15 weeks caused a significant increase in body weight (BW) (*p* = 0.015 in 2^nd^ wk; *p* = 0.0015 in 3^rd^ wk and *p* < 0.0001 during the rest of diet exposure), caloric intake (*p* = 0.0013), fat mass (*p* < 0.0001) and adiposity index (calculated as fat/fat + lean mass) (*p* < 0.0001), with a decrease in % lean mass (*p* < 0.0001) (Fig. 3a–f). Treatment with VCE-004.8 significantly reduced BW gain (*p* = 0.0005 in 1^st^ wk of treatment; *p* = 0.0011 in the 2^nd^ wk; *p* = 0.0088 in the 3^rd^ wk; *p* = 0.0068 in the 4^th^ wk and *p* = 0.031 in the last wk of treatment), fat mass (*p* = 0.029) and adiposity (*p* = 0.042), while it increased % lean mass (*p* = 0.046), without affecting total calorie intake. Modest albeit significant effects of VCE-004.8 on BW gain (*p* = 0.048), % fat (*p* = 0.0056) and lean mass (*p* = 0.031), and adiposity index were detected also in CD mice (*p* = 0.032) (Fig. 3).

**Figure 4:**
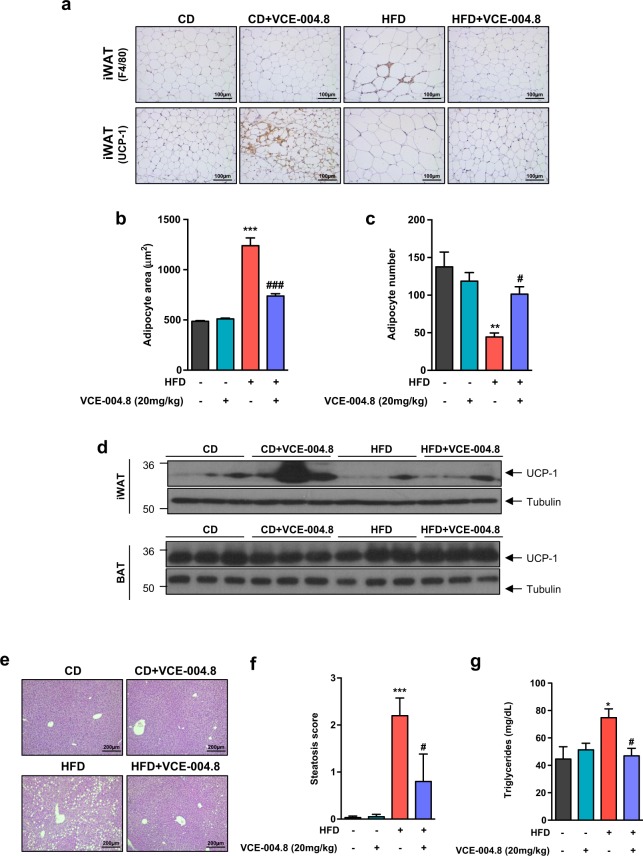
Effect of VCE-004.8 on adiposity and liver steatosis in HFD animal. (**a**) Crown Like Structures (CLS) and browning in iWAT. Representative immuno-histochemistry with anti-F4/80 and anti-UCP-1 antibodies (original magnification × 20, scale bar: 100 μm), (**b**) Adipocyte area (n = 6 animals per group), (**c**) Adipocyte number (n = 6 animals per group). (**d**) Representative Western blot images of UCP-1 protein expression in iWAT and BAT tissues (n = 3). (**e**) H&E-stained liver sections (original magnification × 10, scale bar: 200 μm). (**f**) Steatosis scores (n = 6 animals per group). (**g**) Triglycerides plasma levels (Values correspond to means ± SEM; n = 6 animals per group). ^*^P < 0.05, **P < 0.01 and ^***^P < 0.001 HFD mice vs. control (CD) mice; ^#^P < 0.05 and ^###^P < 0.001 VCE-004.8-treated HFD mice vs. HFD mice (ANOVA followed by Turkey’s test).

Macrophage infiltration of white adipose tissue is implicated in the metabolic complications of obesity. A notable feature of adipose tissue in obese mice is the presence of unique clusters of macrophages that surround dead adipocytes, called crown-like structures (CLS). Therefore, to determine the effect of VCE-004.8 on adipose tissue morphology and inflammation, we analyzed adipocyte size by H&E staining and measured the number of CLS by staining iWAT sections with the macrophage marker F4/80. We observed a significant increase in adipocyte volume that paralleled a reduction in the number of adipocytes per field in HFD animals compared to the CD group. VCE-004.8 treatment prevented HFD-induced adipocyte hypertrophy, normalized adipocyte number and caused a reduction in the presence of CLS (Adipocyte area: *p* < 0.0001 HFD vs CD; *p* < 0.0001 HFD + VCE-004.8 vs HFD; Adipocyte number: *p* = 0.0034 HFD vs CD; *p* = 0.0498 HFD + VCE-004.8 vs HFD; Fig. 4a–c).

Next, to investigate the potential effect of VCE-004.8 on the thermogenic pathway we investigated the adipose expression of UCP-1 by immunochemistry and Western blot. Interestingly, VCE-004.8 induced a clear iWAT browning in control mice, but responses were less clear in HFD mice. Moreover, the expression of UCP-1 in the BAT was not modified after VCE-004.8 treatment, in either control or HFD animals (Fig. 4d).

To assess the effect of VCE-004-8 on lipid metabolism, we next analyzed the degree of fat infiltration in the liver and the circulating levels of triglycerides. As expected, liver histochemistry demonstrated the presence of hepatic steatosis in mice fed HFD (*p* = 0.0006 HFD vs CD; Fig. 4e,f), which displayed also a significant increase in serum triglyceride levels (*p* = 0.030 in HFD vs CD) (Fig. 4g). Treatment with VCE-004.8, did not affect the architecture of the hepatic parenchyma nor altered triglyceride concentrations in CD mice, but significantly reduced hepatic steatosis, and fully normalized circulating triglyceride levels in HFD mice (Hepatic steatosis: *p* = 0.0343 HFD + VCE-004.8 vs HFD; triglyceride levels: *p* = 0.022 HFD + VCE-004.8 vs HFD Fig. 4e–g).

**Figure 5:**
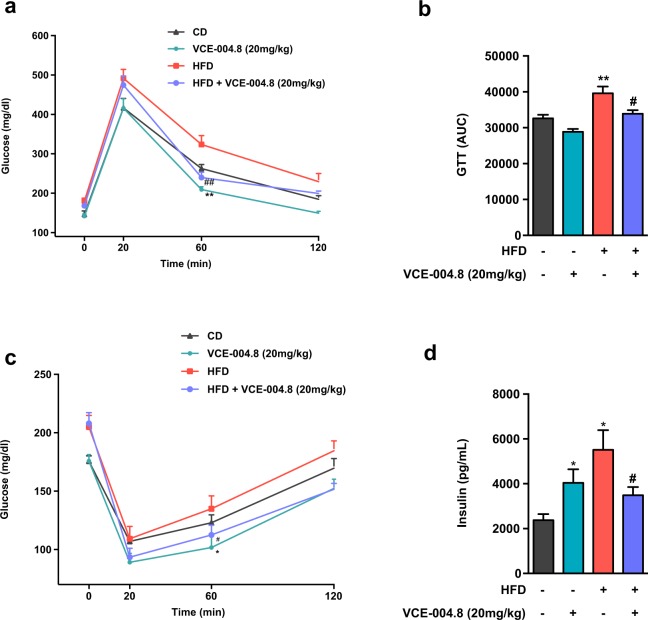
Effect of VCE-004.8 on glucose tolerance and insulin sensitivity. (**a**) Glucose tolerance tests in control (CD) and HFD mice treated with VCE-004.8 or vehicle for three weeks. In addition to time-course profiles, integral glucose responses in GTT are presented in (**b**), calculated as area-under-the-curve (AUC). (**c**) Insulin tolerance tests in CD and HFD treated with VCE-004.8 or vehicle for three weeks. In addition, in (**d**) basal insulin levels at the end of the three-week treatment period are shown for the four experimental groups. Values correspond to means ± SEM of at least 8 mice per group. ^*^P < 0.05 and ^**^P < 0.01 VCE-004.8-treated mice or HFD mice vs. control (CD) mice; ^#^P < 0.05 and ^##^P < 0.01 VCE-004.8-treated HFD mice vs. HFD mice treated with vehicle (ANOVA followed by Turkey’s test).

Continuous exposure of male mice to HFD caused also perturbations in glycemic homeostasis, characteristic of MetS, which were documented by a significant worsening of glucose tolerance, as revealed by GTT (*p* = 0.026) (Fig. 5a,b), and a (moderate) decrease in insulin sensitivity in ITT (Fig. 5c), compared to CD mice. In addition, HFD mice displayed elevated basal glucose (200.74 ± 8.9 mg/dl vs. 153.3 ± 4.73, N = 20/group – measured before initiation of the pharmacological intervention; *p* = 0.012) and insulin levels (*p* = 0.028; Fig. 5d), as evidence for a state of insulin resistance. Treatment of HFD mice with VCE-004.8 significantly improved glucose tolerance, as demonstrated by individual time-course profiles (*p* = 0.0063 in HFD + VCE-004.8 vs HFD at 60 minutes) (Fig. 5a) and integral area-under-the-curve (AUC) responses (*p* = 0.012) (Fig. 5b), and enhanced insulin sensitivity, as documented by ITT profiles and the normalization of basal insulin levels (*p* = 0.028) (Fig. 5c,d). Notably, VCE-004.8 administration to CD mice also tended to improve also glucose tolerance (*p* = 0.0008 at 60 minutes) and insulin sensitivity (*p* = 0.042 at 60 minutes) and significantly increased basal insulin levels in lean animals (*p* = 0.021).

### Effect of VCE-004.8 on metabolic and hormonal markers

**Figure 6:**
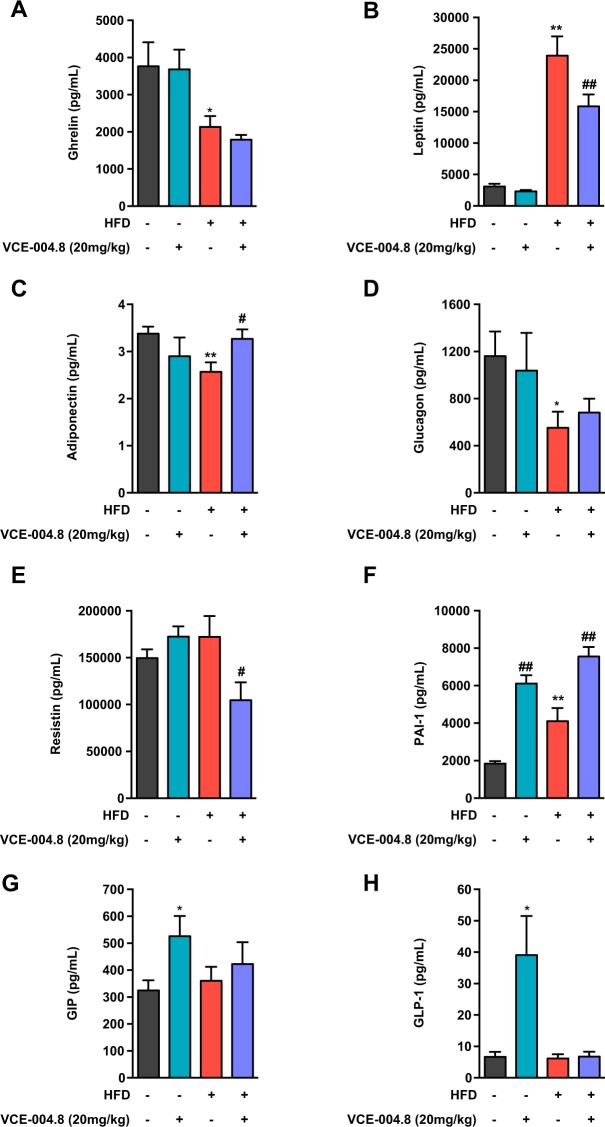
Effect of VCE-004.8 on different metabolic hormones. A panel of hormones and circulating factors, with key roles in metabolic homeostasis, were assayed in control (CD) and HFD male mice, at the end of the three-week period of treatment with VCE-004.8 or vehicle. The factors assayed were: (**A**) ghrelin; (**B**) leptin; (**C**) adiponectin; (**D**) glucagon; (**E**) resistin; (**F**) PAI-1; (**G**) GIP; and (**H**) GLP-1. Values correspond to means ± SEM of at least 8 mice per group. ^*^P < 0.05 and ^**^P < 0.01 VCE-004.8-treated mice or HFD mice vs. control (CD) mice; ^#^p < 0.05 and ^##^p < 0.01 VCE-004.8-treated HFD mice vs. HFD mice treated with vehicle (ANOVA followed by Turkey’s test).

To gain deeper insight into the effects of VCE-004.8 in the context of metabolic disease, a panel of hormonal markers, with pivotal roles in energy and metabolic homeostasis, was assayed in our HFD model. Chronic exposure to HFD resulted in dramatically increased leptin levels (*p* < 0.0001) (Fig. 6B), together with significant reductions in adiponectin (*p* = 0.0058) (Fig. 6C), ghrelin (*p* = 0.0478) (Fig. 6A) and glucagon levels (*p* = 0.035) (Fig. 6D); changes that altogether might contribute to deterioration of the metabolic profile of HFD animals. In addition, HFD mice showed elevated PAI-1 levels (*p* = 0.027) (Fig. 6F), and unaltered concentrations of resistin, GIP and GLP-1 (Fig. 6E,G,H). Treatment with VCE-004.8 partially normalized leptin concentrations (*p* = 0.016), in keeping with the associated reduction of adiposity, and lowered resistin levels (*p* = 0.037), while it increased adiponectin (*p* = 0.028); these joint changes might partially explain the improved metabolic profile of VCE-004.8 treated obese animals. In contrast, no effect on ghrelin or glucagon levels was found after VCE-004.8 treatment in HFD mice. Notably, administration of VCE-004.8 to lean CD animals significantly increased GIP (*p* = 0.027) and GLP-1 levels (*p* = 0.0029), as well as PAI-1 concentrations (*p* < 0.0001). Yet, in HFD mice, the stimulatory effect for incretins (GIP, GLP-1) disappeared, while it persisted for PAI-1 (*p* = 0.0003).

**Figure 7:**
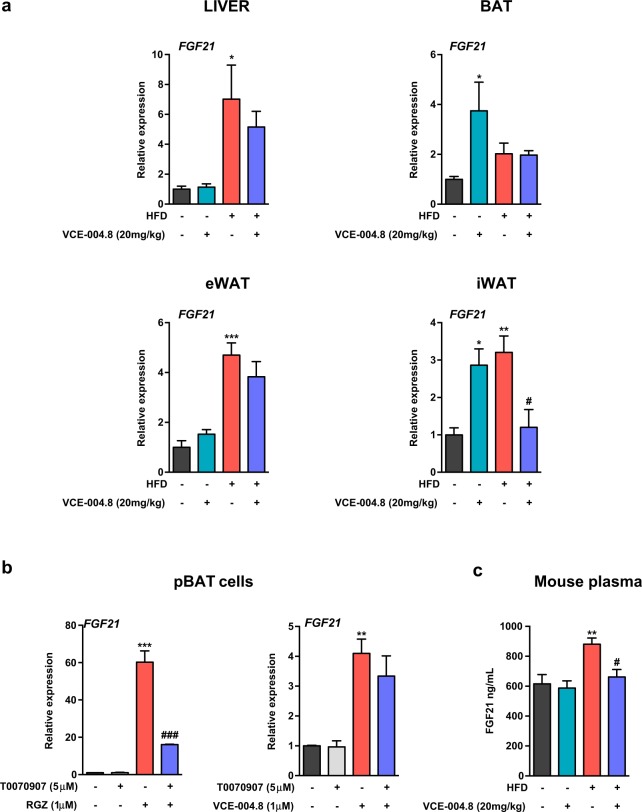
VCE−004.8 modulates the expression of FGF21. (**a**) FGF21 mRNA levels in the liver, BAT, eWAT and iWAT extracted from control and HFD mice treated or untreated with VCE-004.8. Values correspond to means ± SEM of 5–8 mice per group. (**b**) FGF21 gene expression in RGZ- or VCE-004.8-treated pBAT cells in the presence and the absence of T0070907. Results represent the mean ± SD (n = 3). (**c**) FGF21 plasmatic levels were examined using the Mouse FGF21 Quantikine ELISA Kit. Values correspond to means ± SEM of 5–8 mice per group. For (**a**,**c**) ^*^P < 0.05, ^**^P < 0.01 and ^***^P < 0.001 VCE-004.8-treated mice or HFD mice compared to the control mice; ^#^p < 0.05 VCE-004.8-treated HFD mice compared to HFD mice; for (**b**) ^**^P < 0.01 and ^***^P < 0.001 RGZ or VCE-004.8 vs. control; ^##^P < 0.01 RGZ + T0070907 vs. RGZ. Data were assessed by ANOVA followed by Turkey’s test.

Several lines of evidence indicate that the metabolic hormone, FGF21, plays a key role in obesity-associated metabolic syndrome^34^. Indeed, increased plasmatic levels of FGF21 have been found in HFD mice^35^ and metabolically unhealthy obese patients^34^. Since FGF21 can be induced in BAT by PPARγ agonists^36^, we were interested to explore whether or not VCE-004.8 had some role on FGF21 in different tissues and cell types. Our results showed that in control mice, VCE-004.8 enhanced the expression of FGF21 in BAT and iWAT (BAT: *p* = 0.0295 VCE-004.8 vs CD; iWAT: *p* = 0.0301 Fig. 7a), which may reflect the activation of PPARγ by VCE-004.8 in these tissues. Accordingly, VCE-004.8, as well as RGZ, also induced the expression of FGF21 mRNA in the pBAT cell line, although to different extent, which confirms that VCE-004.8 is a PPARγ partial agonist. Interestingly, T0070907 greatly inhibited RGZ-induced FGF21 expression but did not show a significant effect on the expression of this gene mediated by VCE-004.8, highlighting the importance of the alternative binding in the PPARγ LBP for the biological activity of VCE-004.8 (*p* < 0.0001 RGZ vs untreated; *p* = 0.0040 VCE-004.8 vs untreated; *p* < 0.0001 RGZ + T0070907 vs RGZ; Fig. 7b). HFD mice showed an enhanced expression of FGF21 mRNA in liver, eWAT and iWAT tissues that was reduced by VCE-004.8, especially in iWAT. In addition, the circulating levels of FGF21 were significantly increased in HFD, while treatment with VCE-004.8 normalized them (*p* = 0.0095 HFD vs CD; *p* = 0.0382 HFD + VCE-004.8 vs HFD; Fig. 7c).

## Discussion

Obesity and metabolic syndrome (MetS) are reaching pandemic proportions worldwide, and their associated co-morbidities represent an enormous medical and economic burden for our society^37^. The recognition of the multi-factor nature of these diseases and the involvement of numerous deregulated signaling systems in their insurgence have prompted the search of strategies based on poly-pharmaceutical agents that simultaneously target various key pharmacological end-points^38,39^. This polyvalent approach is less prone to the complications associated to multi-drug therapies and has the potential to alleviate the combined deregulation of different factors (food intake, glucose metabolism, energy expenditure) associated to the insurgence of MetS and its complications.

Cannabinoids, as exemplified by ^9^Δ-THC and CBD, are well known and clinically validated multi-target agents, whose biological profile can be modulated by structural modifications. Thus, CBD is a poor PPARγ agonist, unable to bind CB_2_ and to activate the HIF pathway, but oxidation of its resorcinol core increases PPARγ binding, and the introduction of an additional nitrogen substituent beneficially affects chemical stability and induces CB_2_ binding and PHDs inhibitory activity^27,28^. A comprehensive investigation on the biological profile of these compounds (cannabinoid aminoquinones) showed that they could bind to both the canonical and the alternative sites of PPARγ, but also that PPARγ alone was unable to fully recap their biological profile, in accordance with their polypharmacology^40^. Eventually, the benzylamino adduct of the quinone form of CBD (VCE-004.8) emerged from these studies as a potential candidate for pleiotropic pharmacological interventions, and we present compelling evidence that VCE-004.8 has potential for the management of obesity and MetS, being capable to positively affect all the main manifestations of the disease.

Conclusive evidence for the optimal profile of VCE-004.8 in the management of metabolic disease came from our pharmacological studies in a validated murine model of HFD-induced obesity and its associated complications. Indeed, male mice fed on HFD for up to 15-weeks displayed all cardinal manifestations of obesity and MetS, including increased BW, increased calorie intake, increased fat mass and adiposity, hepatic steatosis, elevated serum triglycerides, increased basal glucose and insulin levels, as well as perturbed glucose tolerance and (modestly) worse insulin sensitivity. Chronic treatment with a single daily dose of VCE-004.8 for three weeks was sufficient to revert nearly all of these adverse metabolic alterations, since VCE-004.8 treated HFD-animals showed (*i*) significantly lower BW gain, (*ii*) reduced fat mass and adiposity –together with increased % lean mass-, (*iii*) reduced steatosis, (*iv*) normalized circulating triglycerides and basal glycemia, (*v*) reduced basal insulinemia, and (*vi*) significantly improved glucose tolerance and insulin sensitivity. As a whole, these actions overcome all major complications of obesity and MetS and define an ideal profile for the design of novel pharmacological treatments based on VCE-004.8 or its derivatives.

A beneficial impact on additional hormonal alterations associated to MetS was also observed in the HFD rodent model of obesity and MetS. In addition to basal hyperinsulinemia, mice fed HFD for 15 weeks showed significantly increased leptin and PAI-1 levels, together with lower circulating concentrations of adiponectin, ghrelin and glucagon. While some of these endocrine perturbations might be compensatory, others, such as the enhanced leptin and reduced adiponectin levels, could potentially contribute to the metabolic alterations associated to obesity^41,42^. Notably, repeated administration of VCE-004.8 to HFD mice decreased leptin and increased adiponectin levels; with an overall change in the leptin-to-adiponectin ratio that could rationalize the observation of an improved glycemic profile^42^. Furthermore, VCE-004.8 could also lower resistin levels in HFD mice, further contributing to the decrease of insulin resistance. Interestingly, the body weight loss caused by VCE-004.8 in HFD mice, was not associated to an elevation of serum levels of ghrelin, thus preventing ghrelin-induced compensatory hyperphagic responses, and a worsening of insulin resistance^43,44^. On the other hand, the effect of VCE-004.8 on PAI-1 levels is striking and may reflect the effect of this cannabinoquinone on the HIF pathway activation^45^. Although this observation cannot be underestimated in terms of future clinical development, neither micro-vascular thrombosis episodes nor coagulation alterations have been reported for other PHDs inhibitors in preclinical models or Phase 2/3 clinical trials^24,46^.

In addition to an improved metabolic and endocrine profile in HFD-induced obese mice, VCE-004.8 could also induce *positive* metabolic responses in lean mice fed with control diet, including reduced BW gain, fat mass and adiposity, decreased basal glycaemia, improved glucose tolerance and enhanced insulin sensitivity in the absence of obesogenic challenges. Administration of VCE-004.8 to lean mice enhanced UCP-1 expression in iWAT, with browning and enhanced thermogenesis, and increased basal insulin and incretin (GIP and GLP-1) levels. These beneficial effects on the metabolic profile took place in the absence of detectable changes in calorie intake, suggesting the induction of mechanisms for body weight loss and metabolic improvement independent on feeding. The direct and indirect insulinotropic activity of VCE-004.8 is of special interest and warrants further investigation. In this context, it is worth considering that HIF-1α stabilization mediates insulin secretion by pancreatic β cells^47^ and accordingly we found that VCE-004.8 induced HIF-1α and insulin secretion in the pancreatic acinar-derived AR42J cells (Supplementary Fig. 3). Moreover, GIP and GLP-1 have been target of pleiotropic strategies to improve the management of metabolic syndrome^38,39^. Since the stimulatory effects of VCE-004.8 on GIP and GLP-1 were lost in obese animals, a state of resistance to its incretin-secretagogue actions might be associated to obesity, and the elusive mechanistic bases for this effect are worth further investigation.

Our studies unveiled also that VCE-004.8 influences the gene expression and circulating levels of FGF21, as putative mechanism for some of its metabolic effects. In recent years, FGF21 has emerged as master regulator of body weight, glucose homeostasis and insulin sensitivity^48,49^, it being produced in various key metabolic tissues, including the liver and BAT. Our data document the ability of VCE-004.8 to induce FGF21 expression in BAT and iWAT of lean mice *in vivo*, and to enhance FGF21 mRNA levels in a murine BAT cell line *in vitro*. These findings are in line with previous reports showing that PPARγ ligands can enhance FGF21 expression in adipose tissues^36,48^. Notably, in lean individuals, FGF21 has been shown to increase insulin sensitivity^49^. Thus, the above stimulatory responses may contribute to the improved glycemic profile of lean mice treated with VCE-004.8. Intriguingly, while fasting is known to potently enhance FGF21 levels, as major mechanisms to coordinate adaptive responses to starvation^50^, obesity has been shown to cause also an enhancement of FGF21 levels in rodents and humans^34,35^, suggesting a state of “FGF21 resistance”, defined by elevated FGF21 levels and impaired FGF21 receptor function^51^, which might contribute to the aggravation of metabolic state in conditions of obesity. In good agreement, in our studies, HFD caused an elevation of FGF21 levels, associated to increased FGF21 mRNA expression in liver and WAT, while VCE-004.8 administration normalized serum FGF21 concentrations and iWAT FGF21 gene expression; the latter is compatible with an alleviation of the state of FGF21 resistance linked to obesity. Admittedly, however, the effects of VCE-004.8 on FGF21 appear to be tissue-specific, since it did not enhance FGF21 expression in the liver and eWAT of lean mice nor did it normalize FGF21 mRNA levels in these tissues in HFD animals.

In conclusion, structural modification of the phytocannabinoid chemotype has the potential to reshape their biological profile, emphasizing specific areas of their pleiotropic pharmacological potential. We provide evidence that VCE-004.8 is a selective partial PPARγ agonist lacking adipogenic activity that alleviates metabolic perturbations and inflammatory parameters associated to obesity. VCE-004.8 induced a significant reduction in body weight gain, total fat mass, adipocyte volume, plasma triglycerides levels, and liver steatosis in HFD mice. In addition, VCE-004.8 improved sensitivity to insulin in obese mice and regulated the expression of other metabolic biomarkers. Therefore, our findings establish that specific targeting of PPARγ, CB_2_ and HIF pathway with VCE-004.8 could represent a potential therapeutic approach to obesity and T2D, without the harmful effects on adipogenesis and osteoblastogenesis associated with PPARγ full agonists.

## Conclusions

Structural modification of the phytocannabinoid chemotype has the potential to reshape their biological profile, emphasizing specific areas of their pleiotropic pharmacological potential. We provide evidence that the cannabidiol aminoquinone VCE-004.8 is a selective partial PPARγ agonist lacking adipogenic activity that alleviates metabolic perturbations and inflammatory parameters associated to obesity. VCE-004.8 induced a significant reduction in body weight gain, total fat mass, adipocyte volume, plasma triglycerides levels, and liver steatosis in HFD mice. In addition, VCE-004.8 improved sensitivity to insulin in obese mice and regulated the expression of other metabolic biomarkers. Therefore, our findings establish that specific targeting of PPARγ, CB_2_ and HIF pathway with VCE-004.8 may be a therapeutic approach for the management of obesity and T2D, without the harmful effects on adipogenesis and osteoblastogenesis associated with PPARγ full agonists.

## Material and Methods

### Cell lines

Immortalized murine primary brown adipocytes (pBAT) were kindly provided by Prof Francesc Villarroya (University of Barcelona, Spain). pBAT cells were maintained in DMEM with 10% FBS, 2% HEPES, 20 mM L-glutamine, and antibiotics at 37 °C in 5% CO2 (Klein *et al*., 2012). For differentiation, cells were cultured in 6-well plates (250,000 cells/well) and T3 (1 nM) and insulin (20 nM) were added to the media (Growth media) for 24 hours. Then, IBMX (500 μM), dexamethasone (500 nM) and indomethacin (125 μM) were added to the growth media for 48 h (Differentiation media), before changing back to the growth media with just T3 and insulin for an additional 48 h to allow acquisition of a differentiated morphology. Treatment with RGZ (1 µM) and VCE-004.8 (1 µM) in the presence and the absence of T0070907 (5 µM) started at the same time as the differentiation process. Pancreatic acinar-derived AR42J cells were maintained in Dulbecco’s Modified Eagle’s Medium (DMEM), containing 4.5 g/L of glucose, 0.5 g/L of L-glutamine, supplemented with 10% FBS and 1% (v/v) penicillin/streptomycin (Sigma-Aldrich, USA) at 37 °C in a humidified atmosphere containing 5% CO_2_. Cytotoxicity assays were performed using standard YOYO-1 and MTT methods.

### Transient transfections and luciferase assays

To analyse PPARs transcriptional activities HEK-293T cells were cultured in 24-well plates and transiently co-transfected with either GAL4-PPARγ, GAL4-PPARδ, and GAL4-PPARα vectors together with the luciferase reporter vectors GAL4-luc (firefly luciferase) and pRL-CMV (renilla luciferase) using Roti^©^-Fect (Carl Roth, Karlsruhe, Germany). After stimulation, the luciferase activities were quantified using Dual-Luciferase Assay (Promega, Madison, WI, USA).

### Docking analysis and calculation of theoretical PPARγ-binding affinity

Ligand docking, and binding properties were calculated by using the *AutoDock4*^52^ and the *Vina* software^53^ with the virtual screening tool PyMOL^54^. The receptor models used were the PDB references 3B0R^55^, 4EMA^56^, 5Y2O^57^, and 5LGS^58^. Search space for the docking was set around the binding sites described previously^30,31^.

### Mesenchymal stem cells (MSCs) differentiation

The Reina Sofia University Hospital Review Board approved this study and the procedures followed were in accordance with the ethical standards of the ethic committee from Hospital Reina Sofía (Córdoba, Spain) and with the Declaration of Helsinki. The Hematology Service recruited bone marrow donors. All subjects gave their informed consent so that the bone marrow aliquots extracted for clinical purposes could also be used for mesenchymal bone marrow research. MSCs were seeded in α-MEM containing, 15% FCS, 2 mM Glutamine, 1 ng/ml bFGF and antibiotics. Adipogenic (AD) and osteoblast differentiation (OD) was performed as described elsewhere^59^. Treatments with RGZ and VCE-004.8 were initiated at day 0 of the differentiation process. At day 21, adipogenesis and osteoblastogenesis were analyzed by staining the cell with either Oil Red O or Alizarin Red, respectively. Images captured with the light microscope were analyzed with the ImageJ Software (NIH; Bethesda, MD, USA) and mineralization was quantified by removing the staining solution and absorbance was read at 405 nm.

### Animals and experimental design

Six-week old male C57BL/6 mice, obtained from Charles Rivers Laboratories (l’Arbresle, France), were pair-housed at 20–22 °C, under constant conditions of light (14 hours of light; lights on at 7:00 am), and free access to food (*see below*) and water. All experiments were performed in accordance with European Union guideline and approved by the Animal Research Ethic Committee of Córdoba University (2014PI/025). Procedures were designed to minimize the number of animals used and their suffering. At 8 weeks of age, the animals were randomly assigned in two groups (N = 20) to receive either a standard diet (CD) (A04 SAFE Diets, Augy, France), or high-fat diet (HFD) (45% of calories from fat, D12451 Research Diets, New Brunswick, NJ), for 15 weeks. Body weight (BW) and food intake (FI) were monitored once weekly along the first 12 weeks, and twice a week during treatment period. Additionally, energy intake was calculated in the different experimental groups, using the kcal/g index provided by manufacturer. In order to assess the potential metabolic effects of VCE-004.8, mice were treated by daily intraperitoneal injection of this compound during three weeks, from week 12 onwards, in CD and HFD groups (n = 10/group). Control animals received the corresponding vehicle injections (n = 10/group). Body composition analyses were performed by quantitative magnetic resonance (QMR), using the EchoMRI™ 700 analyzer (Houston, TX, software v.2.0). MRI scans were taken before starting diet exposure, at the start of treatment (week 12; see Supplementary Fig. 2), and at the end of experimental procedures (week 15). At the end of the experiment, mice were euthanized and blood and brown adipose tissue (BAT), white adipose tissue (WAT,) and liver were collected. Tissues were snap-frozen on dry ice and/or fixed in 4% formalin for further analysis of molecular expression and histology, respectively.

### Immunohistochemistry and Western Blots

Liver tissues were processed and 5 μM-thick paraffin-embedded tissue sections were stained with hematoxylin and eosin (H&E). A semi-quantitative score was assigned to evaluate the extent of steatosis according to the Kleiner system (0, <5%; 1, 5–33%; 2, 33–66%; and 3, >66%)^60^. For IHC analysis, 7 μM-thick paraffin-embedded tissue sections of inguinal white (iWAT) adipose tissue were used. Antigen retrieval was performed in 37 °C trypsin (pH 7.8) for 1 h or 10 mM sodium citrate buffer (pH 6) at 95 °C for 10 min. Sections were incubated with F4/80 antibody (1:50; MCA497, Bio-Rad) or UCP-1 antibody (1:500; ab10983, Abcam) overnight at 4 °C, respectively. Then, the slides were incubated for 1 h at room temperature with the appropriate biotin-conjugated secondary antibody (Merck Millipore). Reaction was stained with DAB substrate kit (Merck Millipore), and subsequent counterstaining with hematoxylin and mounting. Samples were analysed with a Leica DM2000 microscope and pictures were taken with a Leica MC190 camera. For Western blots, proteins were isolated from brown (BAT) and inguinal white (iWAT) adipose tissues; 30 μg samples were boiled at 95 °C in Laemmli buffer and electrophoresed in 10% SDS/PAGE gels. Separated proteins were transferred (20 V for 30 min) to PVDF membranes and blocked in 0.1% Tween 20 in TBS solution containing 5% non-fat dry milk for 1 h at room temperature. Membranes were incubated with the UCP-1 antibody overnight at 4 °C (1:2000). For loading control, α-tubulin levels were assayed in the same samples (1:10.000; DM-1A, Sigma,). Membranes were washed and incubated with the appropriate horseradish peroxidase-conjugated secondary antibody for 1 h at room temperature and detected by chemiluminescence system (GE Healthcare Europe GmbH, Freiburg, Germany). To measure the steady state levels of HIF-1α protein in AR42J cells we used the anti-HIF-1α mAb (610959) that was obtained from BD Biosciences (Madrid, Spain).

### Intraperitoneal glucose and insulin tolerance tests, and triglyceride determinations

The animals were ip injected with a bolus of 2 g of glucose per kg BW, after a 5 h period of food deprivation, and blood glucose levels were determined at 0, 20, 60 and 120 min after injection. For ITT, the animals were subjected to ip injection of 1 U of insulin (Sigma Aldrich) per kg body weight, after a 5 h fasting. Blood glucose levels were measured at 0, 20, 60 and 120 minutes. All glucose concentrations were measured using a handheld glucometer (Accu-Check Advantage®; Roche Diagnostics). In addition, serum triglyceride levels were assayed, using a GPO-POD assay kit (Triglyceride Liquid kit 992320, Quimica Analitica Aplicada SA, Spain).

### Determination of hormonal and metabolic markers

Circulating adipokine levels of leptin, insulin, ghrelin, resistin, glucagon, gastric inhibitory peptide (GIP), glucagon-like peptide 1 (GLP-1), plasminogen-activator inhibitor-1 (PAI-1) and adiponectin were measured using quantitative Bio-Plex Pro™ Mouse Diabetes 8-Plex immunoassay (#171F7001M; Bio-Rad Laboratories, Hercules, CA, USA) and Bio-Plex Pro Mouse Diabetes Adiponectin assay #171F7002M (Bio-Rad) according to the manufacturer’s instructions. Additionally, serum FGF21 levels were measured using a commercial ELISA kit (Quantikine MF2100; R&D Systems, Minneapolis), following the manufacturer’s protocol. To determine insulin secretion *in vitro* AR42J cells were stimulated with VCE-004.8 for 24 h and after that the cell were washed and incubated with 1.5 mL of Krebs-Ringer bicarbonate buffer (KRBB) containing 143.5 mM NaCl, 5.8 mM KCl, 2.5 mM CaCl_2_, 25 mM HCO_3_, 0.3% BSA (Sigma) and 3.3 mM glucose at 37 °C for 1 h. Then, cells were washed and incubated with 1.5 mL KRBB buffer with 27.7 mM glucose at 37 °C for 1 h. Total insulin was measured in the supernatants using a rat (10-1250-01) ELISA (Mercodia AB, Uppsala, Sweden).

### Real-time PCR

For gene expression, cells were collected at day 7 (MSCs) or 14 (HPAs) of differentiation and total RNA was extracted using the High Pure RNA Isolation kit (Roche Diagnostics, Switzerland). For tissues, mRNA was extracted using the FavorPrep™ Tissue Total RNA Purification Mini Kit (Favorgen Biotech Corp., Ping-Tung, Taiwan) and Qiagen RNeasy Lipid Kit (Qiagen, Hilden, Germany). For real-time PCR analysis, RNA was reverse transcribed and the HPRT or GAPDH gene was used to standardize mRNA expression in each sample. The primers used in this study are indicated in Supplementary Table S2.

### Statistical analysis

*In vitro* data are expressed as mean ± SD and *In vivo* results are represented as mean ± SEM. Statistical analyses were performed on data distributed in a normal pattern, using Student’s *t* tests or Analysis of Variance (ANOVA). *P* < 0.05 was taken as the minimum level of significance. Statistical analysis was performed using GraphPad Prism^®^ version 6.01.

## Electronic supplementary material

Supplementary data
