# Supplementary material for: VCE-004.8, A Multitarget Cannabinoquinone, Attenuates Adipogenesis and Prevents Diet-Induced Obesity
Source: Sci Rep. 2018 Oct 31;8:16092. doi: 10.1038/s41598-018-34259-0 (PMC6208444; doi:10.1038/s41598-018-34259-0)
Supplement: Supplementary file 1 — Supplementary data [file 41598_2018_34259_MOESM1_ESM.pdf]

VCE-004.8, A MULTITARGET CANNABINOQUINONE, ATTENUATES ADIPOGENESIS AND PREVENTS DIET-INDUCED OBESITY

Belen Palomares[1,2,3,8], Francisco Ruiz-Pino[1,2,3,8], Carmen Navarrete[4], Inmaculada Velasco[1,2,3], Miguel A. Sánchez-Garrido[1,2,3], Carla Jimenez-Jimenez[1,2,3], Carolina Pavicic[5], Maria J. Vazquez[1,2,3], Giovanni Appendino[6], M. Luz Bellido[4,7], Marco A Calzado[1,2,3], Manuel Tena-Sempere[1,2,3,9], Eduardo Muñoz[1,2,3,9,*]

[1]Instituto Maimónides de Investigación Biomédica de Córdoba (IMIBIC), Córdoba, Spain; [2]Departamento de Biología Celular, Fisiología e Inmunología, Universidad de Córdoba, Córdoba, Spain; [3]Hospital Universitario Reina Sofía, Córdoba, Spain; [4]Vivacell Biotechnology España, Córdoba, Spain; [5]Innohealth Group, Madrid, Spain; [6]Dipartimento di Scienze del Farmaco, Università del Piemonte Orientale, Novara, Italy. [7]Emerald Health Pharmaceuticals, San Diego, USA.

SUPPLEMENTARY DATA

Table 1: Primers used in this study

Gene	Forward (5´→3´)	Reverse (5´→3´)
h-PPARγ2	GCGATTCCTTCACTGATACACTG	GAGTGGGAGTGGTCTTCCATTAC
h-LPL	GGCGCTACCTTGAGATAGAGTTCTG	TGTTTTCTACAGGGTGCTTTAGATGAC
h-aP2a	CCAGGAATTTGACGAAGT	TCTCTTTATGGTGGTTGATT
h-CEBPA	CCTTGTGCCTTGGAAATGCAAAC	CTGCTCCCCTCCTTCTCTCA
h-ADIPOQ	CATGACCAGGAAACCACGACTC	CCGATGTCTCCCTTAGGACCA
h-RUNX2	TGGTTAATCTCCGCAGGTCAC	ACTGTGCTGAAGAGGCTGTTG
h-ALP	CCAACGTGGCTAAGAATGTCATC	TGGGCATTGGTGTTGTACGTC
h-HPRT	ATGGGAGGCCATCACATTGT	ATGTAATCCAGCAGGTCAGCAA
m-FGF21	TGTTTGACCGGATCTACACAC	CCCACAAGAGCACTCCAA
m-GAPDH	TGGCAAAGTGGAGATTGTTGCC	AAGATGGTGATGGGCTTCCCG

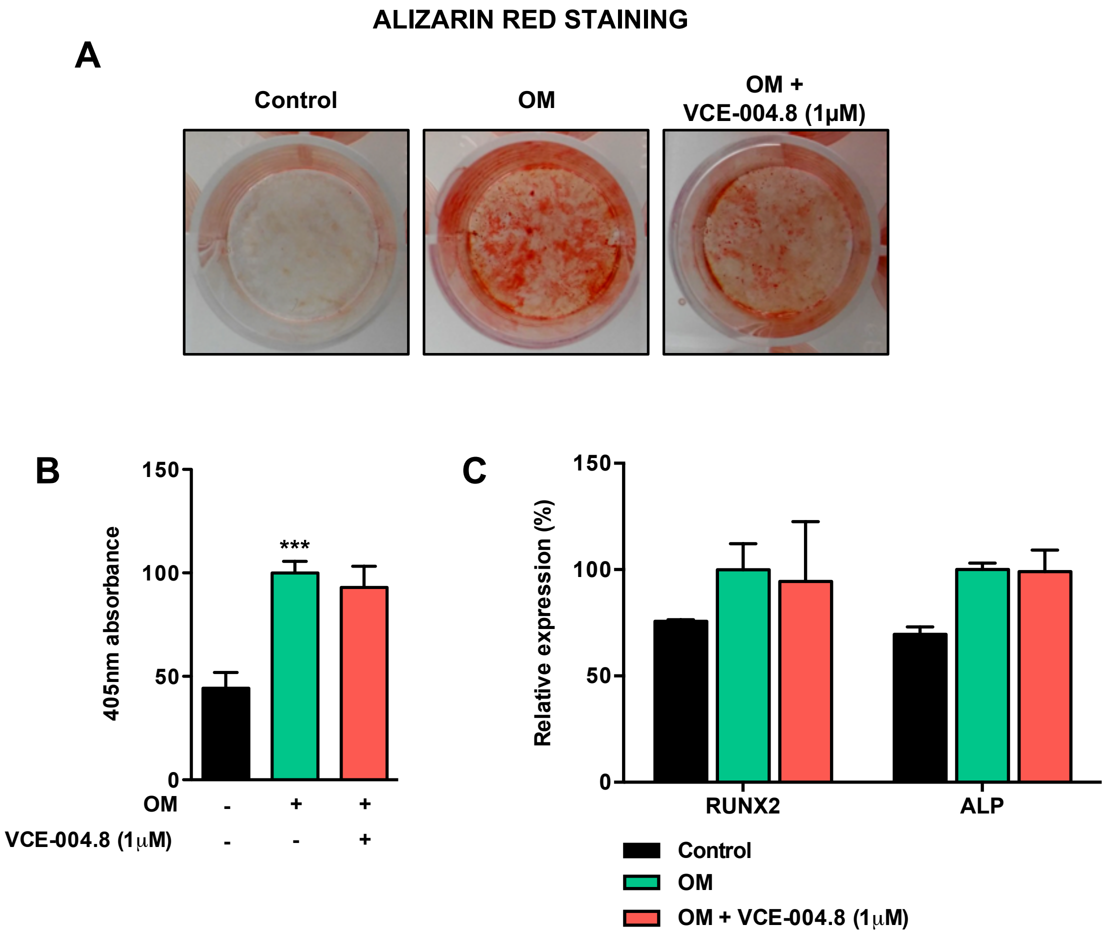

Figure S1. VCE-004.8 does not affect osteoblastogenic differentiation in MSCs.

(A) Alizarin red staining after 21 days of subculture. (B) Quantification of calcium mineral deposits by absorbance at 405 nm. (C) Gene expression of RUNX2 and ALP in MSCs differentiated for 14 days. Data represent the percentage of increase over OM considered as the 100% of osteoblastogenic induction. Results represent the mean ± S.D (n=3). *P<0.05 OM vs. control. (ANOVA followed by Turkey's test).

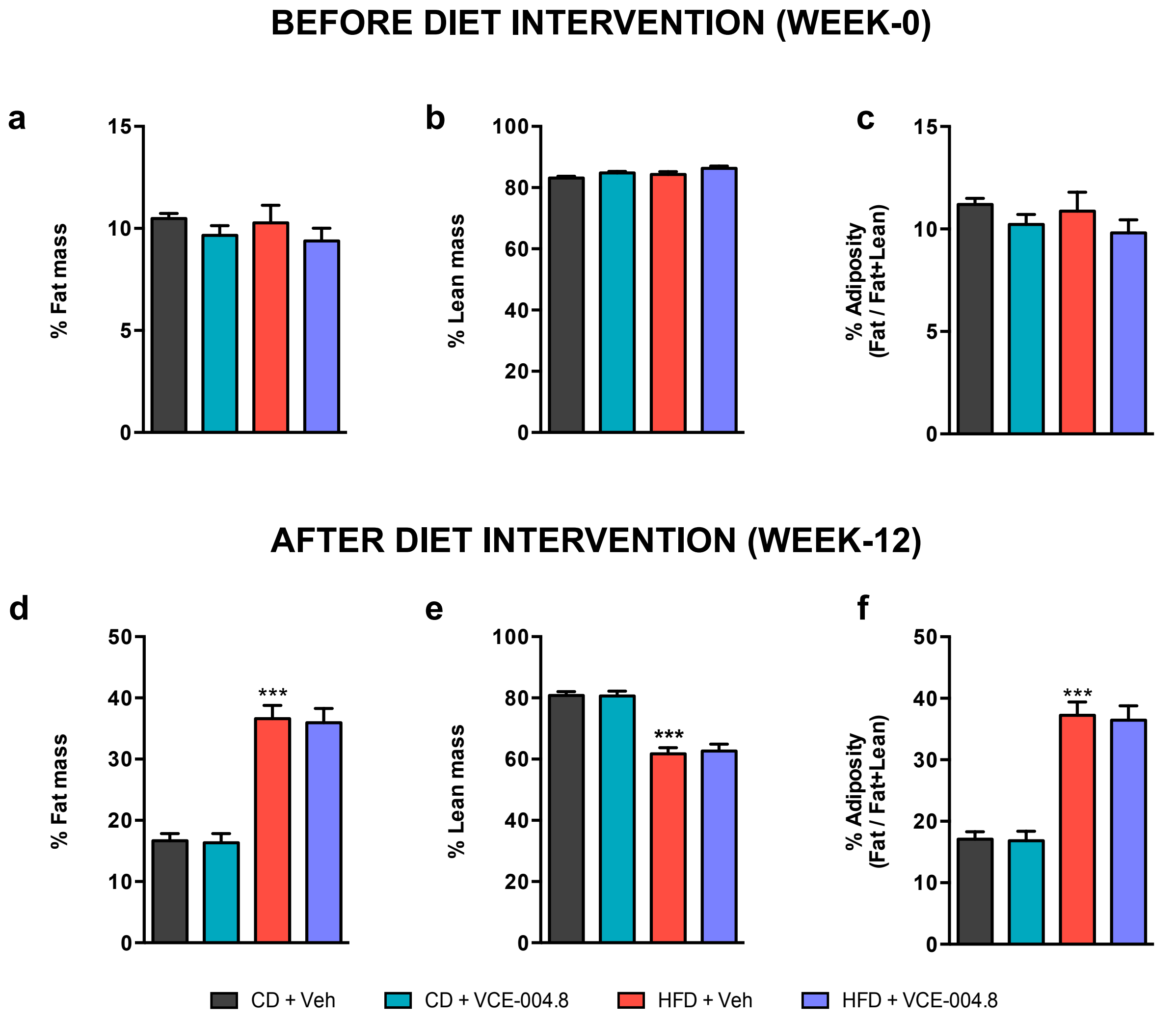

Figure S2. Body composition analysis of experimental animals before initiation of the pharmacological intervention with VCE-004.8

In the *upper panels*, body composition indices are shown from the different experimental groups before initiation of the diet intervention (week-0): % of body fat (**a**), % of lean mass (**b**), and % of percentage of adiposity (calculated as Fat/Fat + Lean mass) are shown. In the *lower panels* (**d-f**), similar indices were recorded after 12 weeks of nutritional intervention with either control diet (CD) or High Fat Diet (HFD), immediately before initiation of pharmacological treatment with VCE-004.8. Note that the animals were divided at the beginning of the study into the four experimental groups: CD + Veh; CD + VCE-004.8; HFD + Veh; and HFD + VCE-004.8, which are presented here to ease comparison with data shown in Figure 3. ***P<0.001 HFD mice vs. control (CD) mice (ANOVA followed by Turkey's test).

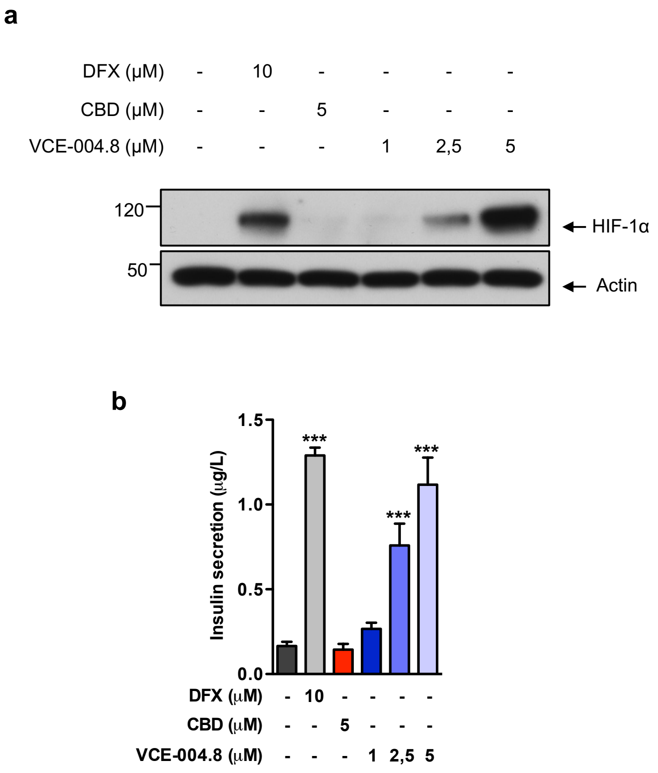

Figure S3. VCE-004.8 stabilises HIF-1α and induces insulin secretion in AR42J cells.

AR42J cells were stimulated with DFX (10 μM), CBD (5 μM) or VCE-004.8 at the indicated concentrations for 6 hours, respectively. Cell lysates were analysed for protein expression by immunoblots (**a**). We show a representative western blot of three independent (**b**) AR42J cells were seeded in 24 well plates, stimulated with the same treatments and incubated with KRBB supplemented with 27.7 mM glucose for 1 h. Insulin secretion was analysed by ELISA. Data are mean ± SD of n = 3 experiments. ***$P < 0.001$ vs. control group (one-way ANOVA followed Dunnett's test).
